# Hematopoeitic Cell Transplantation and CAR T-Cell Therapy: Complements or Competitors?

**DOI:** 10.3389/fonc.2020.608916

**Published:** 2020-12-22

**Authors:** Scott R. Goldsmith, Armin Ghobadi, John F. DiPersio

**Affiliations:** Division of Oncology, Department of Medicine, Washington University School of Medicine, St. Louis, MO, United States

**Keywords:** allogeneic stem cell transplantation, chimeric antigen receptor T cell therapy, cytokine release syndrome, hematologic malignancies, Allo-CAR T

## Abstract

Allogeneic hematopoietic cell transplantation (allo-HCT) and chimeric antigen receptor T cell (CAR T) therapy are the main modalities of adoptive cellular immunotherapy that have widely permeated the clinical space. The advent of both technologies revolutionized treatment of many hematologic malignancies, both offering the chance at sustained remissions for patients who would otherwise invariably succumb to their diseases. The understanding and exploitation of the nonspecific alloreactivity of allo-HCT and the graft-versus-tumor effect is contrasted by the genetically engineered precision of CAR T therapy. Historically, those with relapsed and refractory hematologic malignancies have often been considered for allo-HCT, although outcomes vary dramatically and are associated with potential acute and chronic toxicities. Such patients, mainly with B-lymphoid malignancies, may now be offered CAR T therapy. Yet, a lack of prospective data to guide decisions thereafter requires individualized approaches on whether to proceed to allo-HCT or observe. The continued innovations to make CAR T therapy more effective and accessible will continue to alter such approaches, but similar innovations in allo-HCT will likely result in similarly improved clinical outcomes. In this review, we describe the history of the two platforms, dissect the clinical indications emphasizing their intertwining and competitive roles described in trials and practice guidelines, and highlight innovations in which they complement or inform one another.

## Introduction

The expanding field of immuno-oncology has unlocked the possibility of treating and potentially curing patients with the most life-threatening relapsed and refractory hematologic malignancies. The clinical benefit of allogeneic hematopoeitic cell transplantation (allo-HCT) is mediated by a graft-versus-tumor effect which results from alloreactivity of donor T cells to host major and minor histocompatibility antigens (1–3). Over the past fifty years, we have better understood and refined the process of allo-HCT, improving its success and limiting its complications (4–6). Nevertheless, disease persistence and transplant-related toxicity have driven the necessity for continued innovation.

Now, at the leading edge of immune-oncology, genetically engineered chimeric antigen receptor T (CAR T) therapies promise to advance the treatment of refractory malignancies by combining B-cell-like target recognition with T-cell machinery and memory (7, 8). Notable responses, even among patients who had progressed after allo-HCT, led to the FDA approval of commercial CAR T therapies in young patients with acute lymphoblastic leukemia (ALL), and later in adults with relapsed refractory large cell B cell lymphomas (9). Similar to allo-HCT, disease recurrence after CAR T and treatment-related toxicities require ongoing innovation in product development and toxicity mitigation strategies.

As the novelty and success of CAR T therapies continue to escalate, many now wonder whether CAR T and allo-HCT will continue to coexist and complement one another, or whether some selective pressure, be it cost, convenience, efficacy, or toxicity, will favor only one to persist or to dominate the clinical landscape. At this point, the answer varies depending on the specific disease, practitioner perspective, and even geographic area of practice. Many still view CAR T therapy as a bridge to allo-HCT in patients with ALL, although that stance is not ubiquitous (10). Compare that to multiple myeloma (MM), in which the promise of CAR T efficacy from clinical trials has all but removed allo-HCT from the late-stage MM algorithm, although some centers continue this practice (11, 12).

While at this point it may be impossible to predict whether CAR T or allo-HCT will outlast the other, it is clear that they have been both competitive and complementary. Additionally, lessons have been translated from one platform to the other, such as the management of cytokine release syndrome (CRS), improved efficacy with lymphodepletion, and the potential for “off-the-shelf” allogeneic universal CAR T cells (UCAR T).

In this review, we will provide a historical overview of the two therapies, drawing attention to similarities and differences. We will then analyze the clinical trial data on the interplay between allo-HCT and CAR T therapy and the lessons that have been learned from each. We will describe the knowledge gaps that still exist regarding the sequencing or substitution of one platform with the other, and the ongoing preclinical and clinical work aiming to resolve them. Lastly, we will examine the future directions in which both strategies are heading, emphasizing the indications in which they will be complementary and in which one could out-compete the other.

## Cellular Therapies: The Parallel and Intertwining Histories of Allo-HCT and CAR T

### Hematopoeitic Cell Transplantation and the Birth of Adoptive Immunotherapy

In the middle of the 20^th^ century, preclinical work by Jacobsen, Lorenz, and colleagues gave credence to the concept of the transplantation of bone marrow following lethal irradiation (13, 14). Over the next twenty-five years, numerous physician-scientists sought to translate this to a clinical therapy, initially for radiation-induced aplasia, but subsequently for congenital immunodeficiencies, aplastic anemia, and eventually for acute leukemias (4). Much of the initial clinical work was limited by frustrations and failures. While early reports described the feasibility of allogeneic bone marrow collection, storage, and intravenous infusion into a recipient, little progress was made regarding the impact of histocompatibility differences between donor and host; those with insufficient preparation rejected the graft and those with complete myeloablation often developed profound graft-versus-host disease (GVHD) (15). The development of canine and murine models by Thomas et al. led to a rudimentary understanding of histocompatibility, which they then translated to clinical application. Specifically in hematologic neoplasia, they initially studied syngeneic bone marrow transplantation in a small number of patients who had identical twins. While they observed normal recovery of hematopoiesis, most would relapse (1, 2). Under the hypothesis that the syngeneic immune cells lacked the ability to immunologically target the leukemic cells, they designed a regimen in which the transplant recipients would receive repeated infusions of syngeneic donor lymphocytes along with subcutaneous injections of autologous, lethally-irradiated leukemia cells in order to provide continual antigenic stimulation. This first “immunotherapy” was modestly successful at delaying leukemia relapse, and provided the initial evidence of a graft-versus-leukemia (GVL) effect.

As most patients do not have identical twins, investigators focused on HLA-identical sibling transplantation. As transplant physicians gained experience, refinements in conditioning regimens, improvements in supportive care, and the addition of post-transplant immunosuppression lessened transplant-related mortality and improved survival. One observation was that patients who developed both acute and chronic GVHD were noted to have decreased incidence of relapse, which in some cases translated to improved survival (16). Nevertheless, severe GVHD was often fatal and limited the prospects of allo-HCT, therefore investigators sought to find improved methods of GVHD prevention. T-cell depleted grafts were assessed preclinically and clinically, and while they were associated with reduced GVHD, relapse and graft failure rates were significantly higher negating any beneficial effects (17). This was especially notable in myeloid malignancies, less so in acute lymphoblastic leukemia.

These initial observations stressed the importance of the T-cell mediated GVL effect. With a deeper understanding of the adoptive immunotherapy aspect of allo-HCT, new modifications and therapies were possible. Donor lymphocyte infusions were administered to patients with mixed donor chimerism or early relapse, with durable remissions achieved especially in myeloid malignancies (18–20). Additionally, reduced-intensity conditioning regimens were designed that allowed for older and frailer patients to undergo allo-HCT, with a heavier reliance on the GVL effect (21).

### The Advent of CAR T Therapy

At the same time that nonspecific adoptive cellular therapies (e.g., donor lymphocyte infusions, tumor-infiltrating lymphocytes) were being clinically deployed, novel gene-transfer techniques were allowing for the preclinical *ex vivo* engineering of T-cells harboring CARs. Initially, gene transduction occurred *via* retroviral vectors, but methods involving lentiviral, adenoviral, and non-viral methods would be developed thereafter (7, 22). The initial CAR constructs included an extracellular antigen-specific binding moiety, usually a single chain variable fragment (scFV) of a monoclonal antibody, fused to a transmembrane segment, and an intracellular domain consisting of the CD3ζ signaling domain of the T-cell receptor (TCR) (23, 24). While these first-generation CARs could redirect the specificity of T-cells to target antigens in an HLA-independent fashion, the intracellular signaling of CD3ζ alone lacked the strength to induce proliferation and prolonged antineoplastic activity, resulting in rapid CAR T-cell exhaustion and only modest reduction in tumor growth *in vivo* (25).

In order to achieve the goal of a “living drug”, in which the CAR T cells could continue to proliferate and display persistent antineoplastic activity following the *in vivo* administration, investigators examined methods in which to augment intracellular signaling. Chimeric costimulatory receptors (CCRs) were first developed and introduced into human primary T-cells. Engagement of the CCRs (specifically that with a CD28 intracellular signaling domain) led to increased IL-2 production which allowed for persistence of the T-cells in TCR-activation situations which would otherwise promote apoptosis (26). CD28 and other costimulatory domains, such as 4-1BB (CD137), were then fused with CD3ζ. These “second generation” CAR T cells utilizing one of several potential costimulatory endodomains demonstrated increased persistence, proliferation, and antitumor activity, preclinically (27, 28). In a proof-of-concept clinical pilot, Savoldo et al. treated 6 patients with relapsed non-Hodgkin’s lymphoma who were simultaneously infused with a “first generation” CAR T product harboring only a CD3ζ endodomain and a “second generation” CAR T product harboring both CD3ζ and CD28 endodomains (29). Both had the same CD19-specific scFv exodomain. Peripheral blood examination demonstrated that the second-generation CAR T-cells expanded *in vivo* significantly more in the first two weeks after infusion and persisted longer. Additionally, *ex vivo* engagement of their native TCR could promote their restimulation. In contrast, the first generation CAR T-cells did not expand, could not be restimulated, and did not persist in the infused patients. With expansion and persistence demonstrated in humans, along with efficacy signals in targeting CD19+ B cell malignancies, these second-generation CAR T cells were primed for widespread clinical investigation.

## Clinical Applications of CAR T-Cell Therapy and the Role of Allo-HCT

Current treatment algorithms now incorporate CAR T therapy for specific hematologic malignancies. The initial target for CAR T-cell therapy was CD19, chosen for its broad and high expression on B-cell leukemias and lymphomas, as well as for restriction of its expression to the B-cell lineage which would predict limited off-target effects (30). Theoretically, targeting B-cells would also limit humorally-mediated rejection of the CAR T cells. As such, the CD19 CAR T products would be the first to obtain regulatory approval for B-cell acute lymphoblastic leukemia (B-ALL) in patients up to age 25 and in certain B-cell non-Hodgkin’s lymphomas (NHL). Following a similar path, the CAR-targeting of a lymphoid/plasma cell-restricted surface antigen, B-cell maturation antigen (BCMA), has multiple myeloma on the precipice of at least one approved CAR T product.

How the role of allo-HCT has been impacted by these CAR T therapies is dependent on numerous disease, patient, and therapy factors (**Table 1**). While there is a lack of prospective data addressing the specific intertwining roles of CAR T and allo-HCT, the decisions often require individualized consideration as well as reliance on subgroup data from within existing trials and expert opinion. Hereafter, we dissect such information as it exists for these three disease groups in which CAR T therapy is part of the current treatment paradigm.

**Table 1:** Comparisons of indications and outcomes of allogeneic hematopoeitic transplantation and CAR T-cell therapy.

	Allogeneic HCT	CAR-T
B-ALL		
Indications	High-risk CR1, ≥CR2, Post CAR-T (especially early loss of B-cell aplasia, no prior alloHCT), controversial in active disease	Refractory or 2^nd^ or greater relapse in ≤25 years-old (tisagenlecleucel) Efficacy seen in post alloHCT In clinical development for adult patients: dual-targeting CAR, relapse post CD19 CAR, “off-the-shelf” allogeneic CAR T
CR	N/A	60%–80% (adults) 70%–90% (pediatrics)
OS	30%–60% at 3 years (adults) 60%–75% at 3 years (pediatrics) 20% at 3 years (alloHCT with active disease)	40%–70% at 2 years
Toxicity	aGVHD, cGVHD, graft failure and prolonged cytopenias, infections	CRS, ICANS, prolonged cytopenias, infection
References	(31–34)	See **Tables 2** and **3**
AML		
Indications	Intermediate or unfavorable risk in CR1, ≥CR2, active disease (usually on a clinical trial)	Currently in clinical development for rel/ref active disease CAR Targets: NKG2D, CD123, CLL-1, and CD33
CR/CRi	N/A	50% (early phase I data)
OS	25%–60% at 3 years (adults) 30%–70% at 3 years (pediatrics) 10%–20% at 3 years (active disease)	N/A
Toxicity	aGVHD, cGVHD, graft failure and prolonged cytopenias, infections	CRS, ICANS, marrow ablation (theoretical)
References	(33, 35)	(36–38)
DLBCL		
Indications	Relapse after ASCT – best in patients with >12 mo remission after ASCT, chemosensitive disease, lower NRM with RIC	Rel/ref after two lines of therapy (FDA indications for tisagenlecleucel and axi-cel) Allogeneic CAR T, dual-targeting CAR T, relapse post-CD19 CAR T in clinical development
CR	N/A	40%–60%
PFS	40% at 3 years	Axi-cel: 75% at 2 years in responders, 22% at 2 years in patients with SD Tisagenlecleucel: 83% at 1 year in responders
OS	54% at 3 years	Axi-cel: 50% at 2 years (ITT) Tisagenlecleucel: 40% at 1 year (ITT)
NRM	25%–30%	4% (axi-cel), 0% (tisagenlecleucel)
References	(39)	See **Table 4**
FL		
Indications	Rel/ref FL – better outcomes with chemosensitive disease and RIC	Rel/ref FL (axi-cel, in clinical development)
CR	N/A	80%
PFS	50% at 5 years	50% at 2 years
OS	60% at 5 years	90% at 2 year
NRM	20%	3%
References	(40, 41)	(42, 43)
MCL		
Indications	Rel/ref MCL – better outcomes with chemosensitive disease, RIC, earlier in disease course although controversial	Rel/ref MCL having received at least 2 lines of therapy (brexucabtagene autoleucel, approved indication)
CR	N/A	67%
PFS	40%–50% at 3 years	61% at 1 year
OS	40%–60% at 3 years	83% at 1 year
NRM	15%–25%	3%
References	(44)	(45)
MM		
Indications	Rarely indicated – usually in a younger patient or high-risk disease as part of a clinical trial	In clinical development for triple-class refractory disease and suboptimal response to ASCT
CR	N/A	30%–90%
PFS	30%–40% at 3 years	40%–50% at 1 year
OS	50% at 3 years	75%–90% at 1 year
NRM	20%–25%	2%–5%
References	(12, 46)	(47–49)
		

Allo-HCT, allogeneic hematopoietic cell transplantation; aGVHD, acute graft-vs-host disease; AML, acute myeloid leukemia; B-ALL, B-cell acute lymphoblastic leukemia; CR, complete response; CRS, cytokine release syndrome; DLBCL, diffuse large B-cell lymphoma; FL, follicular lymphoma; MCL, mantle cell lymphoma; N/A, not available; NRM, non-relapse mortality; OS, overall survival; PFS, progression-free survival; PR, partial response; rel/ref, relapsed/refractory.

### Acute Lymphoblastic Leukemia

Treatment of B-ALL has evolved tremendously over the past decade. Prolonged, intensive combination chemotherapy regimens have been very successful at curing a majority of pediatric patients with ALL (50). These pediatric-inspired regimens have been translated to young adult populations, improving relapse-free and overall survival relative to historical comparators, albeit to a lesser extent than that seen in pediatric populations (51). Even some middle-aged and older adults may be cured with front-line chemotherapy, without the need to proceed to allo-HCT.

Concurrently with the advances in therapy, there has been an evolution in the understanding of the clinical and biological heterogeneity of B-ALL. This has allowed for more precise risk stratification based on clinical factors (e.g., age, blast count at diagnosis), cytogenetic/molecular factors (e.g., *BCR-ABL* translocation, Philadelphia chromosome-like ALL, *TP53* alterations with hypodiploidy), and treatment response (e.g., minimal residual disease [MRD] post-induction) (52). Patients with high-risk features are conventionally recommended to proceed with allo-HCT in first clinical remission (CR1) (31, 32, 53). This recommendation is based on observational data suggesting a very high risk of relapse with conventional chemotherapy, and “genetically randomized” prospective trials repeatedly demonstrating a survival benefit in high-risk subsets for those who received HLA-matched sibling allo-HCT.

In both pediatric and adult patients with B-ALL, relapsed and refractory disease carries a dismal prognosis (35). Immediately prior to CAR T therapy, two immunotherapies, inotuzumab ozogamicin and blinatumomab were able to significantly prolong event-free survival and overall survival compared to salvage chemotherapy (54, 55). However, the vast majority of patients in both trials still relapsed and died within 24 months, and long-term survival was achieved only in the minority who proceeded to allo-HCT. Blinatumomab did subsequently establish a niche in converting MRD positive to MRD negative status in patients in CR1 or greater, the majority of whom are bridged to allo-HCT once MRD is no longer detected (56).

The recent advent of CD19-targeting CAR T cells (CART19) provided yet another therapy to the arsenal directed against relapsed/refractory B-ALL. In phase I and II trials, the second-generation CART19 had unprecedented success in achieving remissions in heavily-pretreated patients with B-ALL (**Tables 2** and **3**). With this success, new questions emerged, namely the sequencing of CART19 and allo-HCT, whether CART19 should be used as a bridge to allo-HCT or could be a “destination” in and of itself, and if there were any differences in safety or efficacy in patients who had already undergone allo-HCT prior to CART19. There is yet to be a prospective trial in which patients have been randomly assigned to allo-HCT or observation following CART19, therefore the existing data is limited to an extent by selection of patients fit to undergo allo-HCT post-CART19 and those with a suitable donor. Additionally, there is heterogeneity regarding the length of follow-up and reporting of outcomes following allo-HCT.

**Table 2:** Response and relapse outcomes in trials assessing CD19 CAR-T therapy with CD28/CD3ζ co-stimulatory domains in B-cell acute lymphoblastic leukemia with potential bridging to allogeneic hematopoietic cell transplantation.

	Lee et al. (57)	Park et al. (58)	Curran et al. (59)
Patients, n	51	53	25
Age Category	Pediatric and YA	Adult	Pediatric and YA
Median follow-up, mo	22.5	29	7.7 (28.6 in responders)
Prior allo-HCT, %	35	36	20
CR(MRD^-^), %	61(55)	83(60)	75 (67)
Allo-HCT post-CR,%	75	39	83
Relapse after CR: overall/after allo-HCT %	29/9.5	56/35	33/27

Allo-HCT, allogeneic hematopoietic cell transplantation; CR, complete remissions; MRD, minimal residual disease; YA, young adults.

**Table 3:** Response and relapse outcomes in trials assessing CD19 CAR-T therapy with 4-1BB/CD3ζ co-stimulatory domains in B-cell acute lymphoblastic leukemia with potential bridging to allogeneic hematopoietic cell transplantation.

	Maude et al. (60)	Pan et al. (61)	Gardner et al. (62)	Maude et al. (63)	Hay et al. (64)	Jiang et al. (65)	Frey et al. (66)
Patients, n	30	51	45	75	53	58	35
Age Category	Pediatric and YA (25), Adult (5)	Pediatric, YA, and Adult	Pediatric and YA	Pediatric and YA	Adult	Pediatric, YA, and Adult	Adult
Median follow-up, mo	7	NA	9.6	13.1	30.9	7.7	13
Prior allo-HCT, %	60	0	62	61	43	5	37
CR/CRi(MRD^-^), %	90 (77)	86(81)	89 (89)	81(81)	85 (85)	88 (81)	69 (57)
Allo-HCT post-CR,%	11	60	28	13	40	45	38
Relapse after CR: overall/after allo-HCT %	26/0	24/7	45/18	32/NA (50% relapse-free, 50% unknown after allo-HCT)	49/17	38/9	NA (Landmark analysis for EFS p=0.03)

Allo-HCT, allogeneic hematopoietic cell transplantation; CR, complete remissions; MRD, minimal residual disease; NA, not available; YA, young adults.

There is a lack of consensus regarding which B-ALL patients should proceed to allo-HCT after CART19, and among the clinical trials of CART19 such decisions were usually informed by patient age, institutional practice, and the expected persistence of the CAR T cell product (67, 68). A key determinant of persistence appears to be whether the co-stimulatory domain employed is CD28 or 4-1BB, with the CD28 constructs demonstrating relatively short persistence. In a phase I/II NIH study of a CD28 CART19 in children and young adults, initially 12 patients achieved a MRD negative CR, of whom 10 proceeded to allo-HCT with durable remission, whereas the two transplant-ineligible patients relapsed (69). The study expanded to 53 patients, 51 with B-ALL, and in the long-term follow-up (median 22.5 months), 60.8% achieved CR, 90% of which were MRD negative (57). Twenty-one of the 28 patients with MRD negative CR proceeded to allo-HCT, after which 2/21 (9.5%) relapsed, compared to 6/7 (85.7%) of those in MRD negative CR who did not proceed to transplant. This difference translated to significant improvement in leukemia-free survival (HR 16.9, 95% confidence interval [CI] 3.4-85.1, p=0.0006). In a large adult trial, 53 patients received a CD28 CART19 with 83% CR and 67% MRD negative CRs (58). Median event-free survival (EFS) was 6.1 months and median OS was 12.9 months. Of those who were MRD negative (n=32), half proceeded to allo-HCT and the other half did not. Allo-HCT was not associated with improved EFS or OS, although survival was poor regardless of transplant.

In the initial phase I/II trial of the 4-1BB CART19, tisagenlecleucel/CTL019, out of University of Pennsylvania and Children’s Hospital of Philadelphia, Maude et al. (60) reported that only three of 30 pediatric and young adult patients underwent subsequent allo-HCT while in MRD^-^ remission (60). Nevertheless this remission persisted 7 to 12 months after tisagenlecleucel infusion. A similarly low rate of allo-HCT after tisagenlecleucel was reported in the phase II ELIANA trial of this product in a similar population, in which only eight of 75 patients proceeded to allo-HCT while in remission (63). Two of the eight had MRD positivity and two others had lost B-cell aplasia within 6 months of the infusion. Of those eight patients, four were known to remain in remission at follow-up while the other 4 had an unknown disease status. An updated analysis of ELIANA demonstrated persistence of tisagenlecleucel with ongoing B-cell aplasia in some patients with follow-up for multiple years, which correlated with ongoing remission (70). Survival was unprecedented and irrespective of subsequent allo-HCT. Based on these results, some argue that the unique biology of pediatric B-ALL and persistence of tisagenlecleucel provide the potential for durable remission without the need to proceed to allo-HCT in this population (71).

Not all 4-1BB CART19 constructs have been associated with persistence and prolonged B-cell aplasia in children. In a phase I/II study of 45 children and young adults, Gardner et al. (62) produced 4-1BB CART19 at a defined 1:1 of CD4+:CD8+ cells, achieving 93% (40 of 43) MRD negative CRs among those who received the product (62). Median duration of B-cell aplasia, however, was relatively short, only 3 months. Loss of B-cell aplasia correlated with occurrence of relapse. Eleven patients underwent consolidative allo-HCT, two of whom were MRD^+^ by next-generation sequencing pre-transplant and recurred following transplant. Summers et al. (72) provided an updated analysis of this trial in which there was a suggested benefit in leukemia-free survival from consolidative allo-HCT in transplant-naïve patients after CART19 as well as among patients who lose B-cell aplasia in ≤63 days, even those with a prior allo-HCT (72).

In a phase II study out of Hebei Yanda Lu Daopei Hospital, Pan et al. treated 51 children and adults with a 4-1BB CART19 which led to 85% MRD^-^ CR/CRi in those who entered with active disease and 100% conversion of MRD^+^ patients to MRD^-^ (61). Sixty-percent (27/45) of these patients proceeded to allo-HCT, the majority of which were from haploidentical donors. Following allo-HCT, two died from complications of the transplant and two patients relapsed. Comparatively, nine of the 18 patients who did not proceed to allo-HCT relapsed at a median time of 64 days, although the reasons for foregoing transplant were unclear. Late relapse (90+ days after CART19) was also significantly better among allo-HCT recipients (p=0.023). A more recent Chinese study corroborated such findings in a similarly heterogeneous population (65). Jiang et al. prospectively compared outcomes of 47 4-1BB CART19 recipients who achieved MRD^-^ CR. 21 transplant-naïve patients proceeded to allo-HCT at a median 44 days after CAR-T. Twenty-six patients did not proceed to transplant as three had previous allo-HCT, five were contraindicated, three lacked donor, and the rest chose to forego it for personal reasons. Two patients died of complications from the transplant (chronic GVHD, infection), and two experienced CD19-negative relapse. Overall, consolidative allo-HCT was associated with improved EFS and RFS (p<0.05) although there was no significant difference in OS. Importantly, no patient was precluded from allo-HCT due to CAR T-related toxicities. Although these studies did not differentiate the outcomes based on patient age, they support transplanting all transplant-naïve patients who achieve MRD-negativity with 4-1BB CART19 on the basis of protection from relapse, although survival benefits are unclear.

Among United States trials of 4-1BB CART19 for B-ALL in adults, there is a suggestion that bridging to allo-HCT provides better outcomes than CAR T therapy in isolation. In a pilot study out of University of Pennsylvania, tisagenlecleucel was administered to five patients at a high dose in a fractionated schedule (HDF), all of whom achieved a CR. In the follow-up study, single infusion of a high dose 4-1BB CART19 was complicated by a high incidence of CRS-related death, and a low dose lacked efficacy, therefore the protocol underwent two amendments ultimately settling on HDF for the remaining participants (66). The MRD^-^ CR rate among HDF recipients (n=20) was 90%, with 2-year EFS and OS of 49.5% and 73%, respectively. Efficacy and safety outcomes were notably better in the HDF schedule than either single-infusion schema. Nine of 24 patients who had achieved CR were consolidated with allo-HCT at a median of 2.6 months after CART19. Landmark analysis by allo-HCT demonstrated a significant improvement in EFS (p=0.029) and nonsignificant improvement in OS (p=0.09). Work out of the Fred Hutchinson Cancer Research Center produced similar findings in a trial of 53 adults with B-ALL who received a 4-1BB CART19 (64, 73). 45 patients achieved MRD-negative CRs, of whom eighteen (40%) proceeded to allo-HCT. In univariate analysis, allo-HCT was associated with longer EFS compared to no allo-HCT (HR 0.31, p=0.014), as well as after adjusting for other factors associated with improved EFS.

In summary, until randomized controlled trials address the specific question of allo-HCT after CART19 for B-ALL, the decision to proceed to transplant must be individualized based on key patient, disease, and product factors. Most would argue that recipients of a CD28-based CART19 should proceed to allo-HCT due to lack of persistence (71). Young patients who receive tisagenlecleucel, which to date is the only FDA approved commercial product, may be able to forego allo-HCT, as sustained remissions have been seen in such patients. Prior allo-HCT or extensive prior treatment may also favor avoiding subsequent allo-HCT after CART19. Loss of B-cell aplasia, especially within 6 months of CAR T infusion in patients with B-ALL, likely warrants consideration of allo-HCT among those who initially forego it (74). Some argue that predicting persistence of 4-1BB CART19 is difficult and that relapse could occur due to lack of persistence or secondary to the loss of CD19 on leukemia cells; therefore it is reasonable to offer and prepare for allo-HCT in all patients following CART19 (75). The decision to pursue allo-HCT is only likely to become more obfuscated as CAR T therapies with new and/or multiple targets, improved persistence, and universal allogeneic off-the-shelf CAR T (UCART) are developed and deployed.

### Non-Hodgkin’s Lymphoma

The role of CAR T therapy is actively evolving in the treatment strategy of Non-Hodgkin’s lymphoma (NHL), and varies based on NHL subtype. Likewise, the role of allo-HCT is also in flux for NHL, in large part due to the introduction and dissemination of CAR T therapy. Hereafter, we address the trial data for CAR T therapy based on NHL subtype as well as the dynamic status of allo-HCT in these diseases.

#### Diffuse Large B-Cell Lymphoma

The treatment paradigm for early relapsed or refractory diffuse large B-cell lymphoma (DLBCL) involves salvage chemotherapy followed by autologous stem cell transplantation (ASCT) for those that respond to the salvage regimen, in transplant-eligible patients (76, 77). Historically, allo-HCT consolidation after an initial salvage regimen was associated with decreased incidence of relapse compared to ASCT, but was more toxic resulting in comparable relapse-free and overall survival (78). Studies did posit an immunotherapeutic graft-versus-lymphoma effect that could be exploited in the event of relapse after ASCT or failure to mobilize sufficient stem cells, therefore allo-HCT was relegated to such scenarios (79). A CIBMTR analysis examining allo-HCT in the era immediately prior to the development of novel agents and CAR T therapy highlighted the limited options for and poor prognosis of patients necessitating allo-HCT for advanced DLBCL. Relapse rate was inversely correlated with conditioning intensity, although myeloablative regimens yielded non-relapse mortality of 56%, translating to similarly poor 5-year survival of around 20% (80).

While direct comparisons to allo-HCT are lacking and follow-up is still limited, data from the major trials of CD19 CAR T therapy for relapsed/refractory DLBCL and real-world registries suggest durable CR rates of 30 to 40% with treatment-related toxicities that are more benign and relegated to the acute setting (**Table 4**). In the pivotal ZUMA-1 trial, axicabtagene ciloleucel (axi-cel), a CD19-targeting CAR T cell with a CD28 co-stimulatory domain, yielded an objective response rate of 82% and CR rate of 54% (86). In an updated analysis with a median follow-up of 27.1 months, a significant number of patients had converted from SD or PR at one month to CR by 6 months (84). The estimated 24-month overall survival was 50.5%, and durable remissions were highlighted by an estimated 24-month PFS of 75.0% and 72.0% among those with a CR and PR, respectively. No patient had undergone an allo-HCT prior to axi-cel, and only two patients underwent allo-HCT while responding to axi-cel. Unlike the experience with B-ALL, loss of B-cell aplasia was not a predictor of disease recurrence, and durable responses did not appear to require prolonged persistence of functional CAR T cells. Grade 3 or worse cytokine release syndrome (CRS) and immune effector cell-associated neurotoxicity syndrome (ICANS) occurred in 11% and 32% of patients, respectively, but no deaths were attributed to axi-cel, and most treatment-related toxicities were confined to the peri-treatment period.

**Table 4:** Summary of trials of CD19 CAR-T therapy with B-cell non-Hodgkin lymphoma.

	Schuster et al. (81)	Kochenderfer et al. (82)	Schuster et al. (83)	Locke et al. (84)	Abramson et al. (85)	Wang et al. (45)	Jacobson et al. (43)
Patients, n	28	22	111	108	268	74	87
Disease(s)	DLBCL, FL	DLBCL, tFL, PMBL, MCL, FL	DLBCL	DLBCL, tFL, PMBL	DLBCL, PMBL, FL3B	MCL	FL, MZL
Co-stimulatory domain	4-1BB/CD3ζ	CD28/CD3ζ	4-1BB/CD3ζ	CD28/CD3ζ	4-1BB/CD3ζ	CD28/CD3ζ	CD28/CD3ζ
Median follow-up, mo	28.6	12.5	14	27.1	10.8	13.1	11.5
Prior allo-HCT, %	7	0	0	0	3	0	NA
CR/PR, %	57/7	55/18	40/12	58/25	53/20	59/36	80/15
Allo-HCT post-CR,%	11	4	0 (6 non-responders underwent allo-HCT)	5	NA	1.3	NA
OS	DLBCL: 50% at 22 mo FL: 93% at 28.6 mo	NA	59% at 12 mo	50.5% at 24 mo	mOS 10.8 mo	83% at 12 mo	NA
PFS in CR/PR, %	DLBCL: 43/0 at 28.6 mo FL: 70/0 at 28.6 mo	63/0 at 12 mo	83% at 12 mo	75/72 at 24 mo	mDoR 13.3 mo; mPFS 6.8 mo	61% at 12 mo (all patients)	NA

Allo-HCT, allogeneic hematopoietic cell transplantation; CR, complete response; DLBCL, diffuse large B-cell lymphoma; FL, follicular lymphoma; MCL, mantle cell lymphoma; MZL, marginal zone lymphoma; NA, not available; OS, overall survival; PFS, progression-free survival; PMBL, primary mediastinal B-cell lymphoma; PR, partial response; tFL, transformed FL.

Tisagenlecleucel was examined in the pivotal single-armed, phase II JULIET trial in DLBCL and transformed follicular lymphoma (83). Patients with a prior allo-HCT were excluded. A notably higher percentage of patients had relapsed after a prior ASCT compared to the ZUMA-1 trial. The best ORR was 52%, 40% with CR and 12% with PR, with 43% conversion rate from PR/SD to CR at a median of 2 months post-infusion, but as late as 17 months. The estimated 12-month PFS was 83% among those with a CR or PR, and 12-month OS was 50% for all who received an infusion. Grade 3 or higher CRS or ICANS occurred in 22% and 12% of patients, respectively, although were mainly confined to the 8-week period after infusion. No deaths were attributed to the CAR T product. Similar to axi-cel, many patients had loss of B-cell aplasia although this was not associated with disease recurrence. Five non-responders proceeded to allo-HCT, although none of the patients proceeded to allo-HCT while experiencing a response to CAR T.

These two pivotal trials led to the commercial approval of axi-cel and tisagenlecleucel for DLBCL relapsed after or refractory to at least two lines of therapy. They demonstrated notable efficacy in a population of patients with historically poor outcomes, even in those with chemorefractory disease or high-risk features. In comparison, chemosensitivity is usually a prerequisite for proceeding to allo-HCT as chemorefractoriness portends a high risk of relapse in this setting. Such findings were corroborated by real-world reports of CD19 CAR T for DLBCL, with comparable efficacy and an improved safety profile, in part due to the learned management of acute toxicities (87, 88). With comparable or improved efficacy relative to allo-HCT and toxicities that appear generally more tolerable and limited in duration, CAR T therapy has likely supplanted allo-HCT for the treatment of multiply relapsed DLBCL.

#### Mantle Cell Lymphoma

Mantle cell lymphoma, although rare, is uniquely challenging in that it invariably relapses following initial therapy with induction and ASCT consolidation, its clinical course is often aggressive, and it frequently becomes refractory to chemotherapy and novel agents. Although novel therapies such as Bruton’s tyrosine kinase (BTK) inhibitors have prolonged survival, progression is often inevitable and associated with poor survival (89). Allo-HCT has been offered as a potentially curative option with the advantages of providing an uncontaminated graft with theoretical GVL effect, although the advent of numerous targeted agents has been providing longer responses, and patients are more heavily treated at the point of allo-HCT consideration. Reduced-intensity conditioning (RIC) regimens have been favored due to the usual advanced age and comorbidities of MCL patients. In a retrospective registry study by the European Bone Marrow Transplant Lymphoma Working Party, MCL patients undergoing allo-HCT with RIC experienced 1-year NRM of 24%, with long-term disease-free survival of 30% at 4 years (90).

Brexacabtagene autoleucel (KTE-X19) recently garnered accelerated regulatory approval for the treatment of relapsed/refractory MCL. KTE-X19 is the same construct as axi-cel, with a CD28 co-stimulatory domain, although it undergoes a manufacturing process that selectively removes circulating CD19-expressing malignant cells to prevent premature CAR T activation (45). In the ZUMA-2 trial, 74 patients who had relapsed after chemotherapy (anthracycline or bendamustine), an anti-CD20 monoclonal antibody, and BTK inhibitor were treated in a single arm, multicenter phase II trial of KTE-X19, with dosing based on the established dose of axi-cel. The ORR was 85% with a CR rate of 59%, with responses comparable across high risk subgroups. Among those with an initial PR or SD, 57% improved to CR. The 12-month estimated PFS was 61% and OS was 83%. Grade 3 or higher CRS occurred in 15% and ICANS in 31%. One patient had grade 4 cerebral edema. Two deaths occurred relating to the conditioning chemotherapy. One patient proceeded to allo-HCT while in a PR. There are no data comparing allo-HCT with CAR-T in these patients and we currently lack long term follow-up after CAR-T to assess true long term PFS. If CAR-T results in 30% or higher long term disease free survival, in light of lower NRM, it will likely be considered superior to allo-HCT in these patients.

#### Follicular Lymphoma

Follicular lymphoma (FL) is the second-most common NHL and the most common indolent lymphoma. Patients have a variable course, but generally the disease is considered incurable and many patients receive multiple lines of therapy during the course of their disease (91). While not all patients require upfront therapy, those with early treatment failure (disease progression within 24 months of chemoimmunotherapy or 12 months of rituximab monotherapy) have worse outcomes with standard therapy, and warrant more aggressive treatment (92). In the absence of transformed disease and in those who are eligible for transplantation, many will undergo salvage chemoimmunotherapy with the intent to undergo high-dose chemotherapy with ASCT if a CR is achieved. In retrospective studies, ASCT has been associated with prolonged survival compared to salvage chemo-immunotherapy alone (93). Matched-sibling donor allo-HCT provides comparable long-term survival to ASCT, with a significantly lower-risk of relapse but higher upfront NRM (94). Of those patients that survive beyond 24 months, survival was shown to be superior in those who received allo-HCT. In general, nonmyeloablative and RIC regimens are used, some incorporating immunotherapy or radioimmunotherapy (40, 95). Long term PFS may be as high as 70-80% in highly-selected patients. Anecdotally, however, the increased arsenal of novel and investigational therapies for FL, including CAR T cells, is decreasing the use and utility of allo-HCT.

Small prospective trials of CD19 CAR T cells demonstrate the promise of CAR T therapy among patients with relapsed/refractory FL, prompting ongoing larger clinical trials. Among patients treated in the initial prospective case series study of CTL019/tisagenlecleucel out of the University of Pennsylvania, 14 patients who received treatment had advanced FL, 8 of whom had double-refractory disease, three who had undergone prior ASCT and one with a prior allo-HCT (81). 10 of 14 (71%) of patients had a CR at 6 months and remained in remission at a median of 29.3 months. 70% were progression-free at 28.6 month, and 89% who responded maintained the response by the median follow-up period. Severe CRS occurred in five patients (18%) and severe ICANS occurred in three (11%), one case being fatal.

Hirayama and colleagues from the Fred Hutchison Cancer Institute included 8 patients with multiply-relapsed FL (3 with a prior ASCT and 1 with a prior allo-HCT) in their phase I/II study of CD4:CD8 ratio-defined CD19 CAR T (96). Seven (88%) achieved a CR, all of whom remained in remission and one of whom proceeded to allo-HCT. The eighth patient had SD and subsequently underwent radiation therapy with no progression at 36 months. A notable criticism was the high dose of cyclophosphamide that patients received as part of lymphodepletion (97). Importantly, while CRS and ICANS occurred in 50% of patients, no severe adverse events were reported. Axi-cel and tisagenlecleucel are actively being studied in multicenter phase II trials in FL in the ZUMA-5 and ELARA studies, respectively (42). Results from the interim analysis of ZUMA-5 reported an ORR of 95% and CR rate of 80% among 80 patients with FL. With a median follow-up of 11.5 months, 68% of patients had ongoing responses. CRS and ICANS occurred in 11% and 19% of patients (43). While the high response rates and manageable toxicities are promising in multiply recurrent FL, the length of follow-up in these trials is limited. It is therefore unknown whether CAR T therapy will compare favorably or unfavorably with allo-HCT with RIC. Although the novelty of CAR T therapy may lead physicians to lean toward it, this is an area that deserves long term analysis as CAR T should provide durable PFS of 70% or better in order to be a competitive substitute for alloHCT.

#### CAR T Therapy and the Waning Role of Allo-HCT in NHL

As the clinical trial data for CAR T therapy in NHL mount, the role of allo-HCT becomes more questionable. In large part, the toxicities related to CD19 CAR T therapies are acute, limited in severity, and manageable, which makes them more appealing compared to the potentially long-lasting infectious and GVHD complications seen in allo-HCT.

Unlike B-ALL, current evidence does not support consolidative allo-HCT for NHL patients responding to CAR T (98). Additionally, as responses may be delayed and evolve over a prolonged duration, active observation is generally recommended even in patients with a PR or SD post-CAR T. Patients with SD, however, are less likely to achieve a subsequent remission. Therefore, individualized consideration may be given to allo-HCT prior to progression based on the extent of disease, donor availability, and other patient-specific factors. While loss of B-cell aplasia may trigger pursuit of allo-HCT in B-ALL, it has not been associated with disease recurrence in DLBCL, and therefore should not be considered a decision point for transplant (68).

Whether allo-HCT has a role following NHL progression after CAR T therapy is also a point of controversy. While theoretically it would be the principal option that could lead to a durable remission, in practicality it is difficult to achieve a remission pre-transplant in such patients that would justify pursuit of allo-HCT. Additionally, allogeneic transplantation likely eradicates the CAR T cells, which could otherwise potentially be stimulated through a variety of investigational methods in order to attempt to attain a response. Lenalidomide, PD-1 inhibitors, and the bispecific CD3-CD20 monoclonal antibody mosunetuzumab have all demonstrated the potential to recapture a response in patients who progressed after CAR T therapy (99–103). Therefore, pursuit of a clinical trial or off-label use of such agents may be preferred over or should be considered before proceeding with allo-HCT based on the respective risk-to-benefit ratios in such heavily pre-treated patients. As it pertains specifically to bispecific antibodies in NHL, their ease of use and promise of efficacy positions them competitively with CAR T therapy, highlighting evolving dilemmas of patient selection and sequencing of novel immunotherapies.

There is limited experience with CAR T after allo-HCT in NHL as such patients were excluded from larger clinical trials. However, a number of small reports demonstrate that it is safe and feasible to construct donor-derived CAR Ts or pseudo-donor-derived CAR Ts (104–107). In such studies, severe and active graft-versus-host disease (GVHD) was a key exclusion criterion and, while GVHD developed or worsened in a few patients, the severity was mild. Further study is needed in larger homogenous populations to determine if the safety and efficacy of donor-derived CAR T therapy is comparable.

In summary, whereas CD19 CAR T arguably has a complementary role in bridging to allo-HCT in the B-ALL algorithm, it may supplant allo-HCT in most patients with relapsed NHL based on favorable toxicity and at least comparable efficacy. Longer follow-up is needed in most of the NHL CAR T trials in order to confirm this implication.

### Multiple Myeloma

While therapy for multiple myeloma (MM) has dramatically improved over the past two decades, it is generally considered incurable and most patients will die of their disease (108). During the 1990s and early 2000s, during which time novel therapies (i.e., proteasome inhibitors, immunomodulatory drugs, monoclonal antibodies) were in clinical development, allo-HCT was studied in the treatment of MM in several fashions (12). Several studies evaluated ASCT followed by RIC allo-HCT compared to tandem ASCT (109–112). Two meta-analyses of such studies yielded no differences in OS but a significantly higher risk of NRM (113, 114). Allo-HCT as a salvage therapy after relapse has been shown to provide a PFS benefit without OS benefit for a small percentage of patients, as reported in a number of retrospective series and registry studies, and outcomes have been comparable or worse than salvage ASCT in selected patients (12, 115, 116). Therefore, consensus guidelines recommend the use of allo-HCT in these settings only in the context of well-designed clinical trials (117, 118). Interestingly, there are a number of clinical trials ongoing combining allo-HCT with novel therapies as consolidation and maintenance, which may shift the paradigm at a later date. However, they are contending with the ongoing development of CAR T therapy for MM.

B-cell maturation antigen (BCMA) is a cell-surface antigen found on some mature B-cells and normal plasma cells, malignant plasma cells, and importantly not expressed on hematopoeitic stem cells, non B and plasma cell hematopoietic lineages or non-hematopoietic tissue. The first-in-human trial of a BCMA CAR T therapy with a CD28 costimulatory domain demonstrated promising anti-myeloma efficacy (119). The two patients receiving the highest dose level of 9 × 10^6^ CAR T cells/kg body weight had at least a very good partial response, although both had severe CRS and prolonged cytopenias.

In a single-center, phase I, dose-finding clinical trial of a 4-1BB BCMA CAR T therapy, the overall response rate was 12 (48%) of 25 heavily pre-treated MM patients (120). Median duration of response was 124.5 days and 3 patients had durable responses at the time of reporting. Eight (32%) had grade 3+ CRS, one who died of candidemia following prolonged therapy for CRS, and three (12%) had grade 3+ ICANS.

The safety and preliminary efficacy of the BCMA CAR T using 4-1BB costimulatory endodomain, bb2121/idecabtagene vicleucel (ide-cel), was studied in a multicenter phase I trial (121). The ORR among 33 patients was 85%, with a 45% CR/sCR rate and a median PFS of 11.8 months. The follow-up pivotal phase II KarMMa trial of ide-cel yielded a 73% ORR and 31% CR/sCR rate with a median PFS and duration of response of 8.6 and 10.6 months, respectively, and low rate of grade 3+ CRS (5%) and ICANS (3%) (122). Based on these data, regulatory approval for ide-cel in refractory MM patients who have failed at least three independent lines of therapy is actively being pursued.

The LCAR-B38M and JNJ-4528 CAR T therapies are identical constructs comprised of a 4-1BB costimulatory endodomain and two BCMA-targeting single-domain antibodies targeting distinct BCMA epitopes. In the Chinese phase I LEGEND-2 study of LCAR-B38M, 57 patients were infused with 3 split infusions (123). Seven percent had grade 3 CRS, only one patient had ICANS. The ORR was 88%, with a 74% CR rate. Of those with CR, 39/42 were MRD-negative. The 18-month OS was 68%, with a median duration of response of 22 months, 27 months in those with CR. Another smaller trial of the LCAR-B38M products examined 17 patients with high risk features (i.e., extramedullary disease, poor cytogenetics, triple-class refractoriness). While initial responses were promising (88% ORR), factors such as extramedullary disease and the development of anti-CAR T antibodies were associated with relapse, and the 12-month PFS was only 53% (124). The phase Ib/II CARTITUDE-1 study in the United States of JNJ-4528 is ongoing with a 100% ORR and 76% sCR in the first 29 patients and similarly tolerable safety profile, albeit one delayed death from sequelae of grade 4 CRS (47, 48).

The BCMA CAR T platforms are the best positioned to break into clinical practice in the near future. Other CAR targets such as CS-1, immunoglobulin kappa light chain, and CD138 are being explored (11, 125, 126). Additionally, ongoing clinical trials are focused on the appropriate sequencing of BCMA CAR T, specifically addressing whether it should be deployed earlier in patients who experience a suboptimal response from induction therapy and ASCT or frontline for those with high-risk features. Follow-up for BCMA CAR T-treated patients within these trials is still maturing, so it remains unclear whether a durable remission, as seen in some B-ALL and NHL patients, can be expected. The historically inconsistent survival and NRM outcomes in allo-HCT, combined with the substantial treatment burden experienced by the typical MM patient, suggest that this practice will likely be replaced by CAR T therapy, should it deliver on its promise of high responses and some durable remissions. It is unlikely that CAR T will be used as a bridge to allo-HCT, and none of the trials have reported such a practice in any participant.

## The Expanding Frontier of CAR T and Allo-HCT

### CAR T and Allo-HCT in Myeloid Malignancies: An Inseparable Fate

The most common indications for allo-HCT in adults are acute myeloid leukemia (AML) and myelodysplastic syndrome (MDS) (127). Despite the toxicities associated with the transplant itself, relapse remains the most significant cause of treatment failure and death after allo-HCT, highlighting the need for further disease-modifying innovation without added toxicity (128–130). Unlike B-cell malignancies, to date most CAR target antigens for myeloid malignancies have significant overlap with normal myeloid cells and hematopoietic stem/progenitor cells (HSPCs), the eradication of which would likely be poorly tolerated (131). Some of these antigens have been targeted in early clinical trials of CAR T cells with variable toxicity and success thus far, reviewed in detail in Mardiana and Gill (36).

CD123 is one such antigen already being targeted by other investigational immunotherapies (132). Preclinical work suggests that it is a viable CAR target for AML, although it could lead to myeloablation requiring allo-HCT rescue. It is also expressed on vascular endothelium, heightening the risk for toxicity such as capillary leak syndrome (133–135). One risk mitigation strategy has been the production of transient CAR T cells infused serially, a concept that was safe and feasible in a pilot study, but which was discontinued during phase I (136). An ongoing study at the City of Hope Medical Center incorporates a truncated epidermal growth factor receptor (EGFRt) into second-generation CAR T cells, allowing for inactivation with EGFR monoclonal antibodies (37). Early clinical activity has been reported, allowing two of six AML patients to proceed to a second allo-HCT after achieving CRs. Unexpectedly, myeloablation by the CD123 CAR T was not observed as hypothesized, although the intent was to bridge to allo-HCT. A “compound” CAR T cell, with two complete CAR constructs targeting those two antigens connected by a cleavable linker, was generated by Liu et al. and demonstrated promising efficacy in a phase I dose-escalation, with seven of nine participants achieving MRD-negative CR (38). Notably, given the overlap of these targets with normal hematopoietic stem cells, all experienced Grade IV pancytopenia, and six of seven responders went on to alloHCT.

As antigen overlap remains a significant challenge and the immediately perceived role of AML CAR T therapy is as a bridge to allo-HCT, one novel concept is that of genetically engineering an allograft to remove the target antigen from the normal hematopoietic system, and transplanting this allograft in sequence with donor-derived CAR T cells against the specific antigen (131). Kim et al. pioneered the preclinical work in murine and non-human primate models in which they first demonstrated that knock-out of CD33 in the donor hematopoietic stem and progenitor cell population resulted in normal hematopoiesis and myeloid function and normal multilineage engraftment (38). Subsequent administration of CART33 targeting CD33 was able to effectively eliminate CD33+ leukemia without notable off-target effects. Immunotherapeutic targeting of CD33 is already an approved AML therapy (gemtuzumab ozogamicin) with toxicity relating predominantly to the chemotherapy payload (137). The question remains as to how post-transplant immunosuppression will impact the persistence and efficacy of the donor-derived CAR T cells in this platform. Although it has yet to be clinically developed, it is hoped that the concept of combining CAR T therapy with genetically engineered allo-HCT will lead to a synergistic effect on AML with limited added toxicities.

Other approaches to improve the specificity of T-cell therapies for AML are in preclinical and clinical development, including dual-targeting CAR T cells that identify surface antigen combinations that are unique to leukemic blasts as well as T-cell receptor engineered (TCR) T cells that allow for the recognition of intracellular proteins specific to AML blasts (138–140). The existing perspective, however, is that the clinical advances to come from AML cellular therapy will likely need to be combined with allo-HCT in order to achieve the best outcomes. As more is learned about these complementary platforms, lessons from each are likely to benefit one another. 

### Allogeneic “Off-the-Shelf” CAR T Therapy

Numerous challenges to the widespread implementation of autologous CAR T therapy have been described (141). The products are generated from a patient’s autologous T cells, which requires extensive and costly collection and manufacturing efforts. This process is time-intensive, and during the intervening period some patients have difficulty with disease control or complications from bridging chemotherapy. Additionally, the extensive pretreatment brings into question the potency and exhaustion of the cellular therapy.

Due to these limitations, numerous institutions and companies are actively developing “universal off-the-shelf” CAR T products derived from allogeneic sources (UCART), which overcome some of these hurdles although introduce new ones. Principally, alloreactivity can lead to rejection of the UCART mediated by the recipient T and NK cells, and alloreactivity from the UCART can lead to GVHD (142). Numerous studies of graft rejection and GVHD in the context of UCART have demonstrated the role of the T cell receptor (TCR) in recognizing non-self major histocompatibility complex (MHC) molecules and/or MHC molecules complexed with peptides, conferring alloreactivity (143–145). As such, the fundamental understanding of both of these concepts, and methods to mitigate them, are derived from decades of study and observation in allo-HCT.

A unique clinical development in allo-HCT that has translated to preclinical work in the UCART space involves the isolation and therapeutic exploitation of virus-specific T cells that have a limited TCR repertoire. Initially, such allogeneic virus-specific T cells were used in allo-HCT recipients to treat and prevent severe viral infections (146–148). Despite HLA mismatches between the cellular therapy and patients, *de novo* GVHD did not occur with any significant frequency. Therefore, such virus-specific T-cells are currently being bioengineered to harbor CARs for CD19 and other targets (149, 150).

With knowledge of the TCR as the main mediator of both rejection and GVHD, disruption of the TCR through one of a number of gene editing techniques has become the predominate means of preventing GVHD by UCART. In the initial preclinical work, Torikai et al. (151) demonstrated the feasibility of knocking out the gene for the T cell receptor constant α chain (*TRAC*) using zinc finger technology in CD19 CAR T cells, without impairment of their antitumor activity (151). Subsequent methods have employed transcription activator-like effector nuclease (TALEN) technology to develop UCART products, knocking out not only the TCR but also CD52 in the products, allowing for alemtuzumab-based extended lymphodepletion in order to enhance UCART engraftment and persistence and to mitigate UCART rejection without impacting the anti-tumor efficacy of the UCART product itself. Clinical trials of UCART are ongoing in multiple hematologic malignancies (142). The advent of CRISPR/Cas9 technology has allowed for both precision knockout of *TRAC* as well as T cell-specific antigens (e.g., CD7), allowing for possible deployment in T cell ALL without the risk of fratricide (152). Another novel approach that allows for efficient production of UCART products involves adeno-associated virus (AAV)-mediated transduction of the CAR transgene into the *TRAC* locus. This process exploits a site-specific endonuclease and homology-directed repair to simultaneously knock out the native TCR and allows for the CAR to be expressed under the usual transcriptional control of *TRAC* (153, 154). Many of these UCART technologies are in clinical development and, if successful, are poised to make CAR T therapy more accessible and affordable. How they will impact the landscape of CAR T and allo-HCT remains to be seen.

## Discussion and Conclusions

Allogeneic hematopoietic cell transplantation and chimeric antigen receptor T cell therapy are the two principal cellular therapies that have widely permeated the clinical space outside of clinical trials, and remain the focus of many ongoing investigations. They span the spectrum of target specificity which, in part, predicts their efficacy and toxicity. Whether the two modalities complement or compete with one another depends substantially on the disease and the patient and requires a nuanced and individualized approach. For many histologic subtypes of NHL and for relapsed refractory MM, CAR T therapy appears to provide comparable or improved outcomes to allo-HCT with potentially less long-term complications and a chance of durable remissions as a destination therapy. However, allo-HCT already had very niched indications within these diseases secondary to substantial improvements in novel therapies, so likely there will continue to be a role for allo-HCT in select patients, albeit diminished. In B-ALL much of the evidence supports CAR T therapy as a complement serving as a bridge to allo-HCT, especially in adults. However, some patients, especially pediatric patients, may enjoy sustainable remissions with CAR T alone, with active observation for loss of CAR T persistence replacing the immediate need to proceed to transplant while in remission. Should CAR T therapy become a viable treatment option for myeloid malignancies, based on current research there is a high probability that it will be used in conjunction with allo-HCT due to the antigen overlap between malignant myeloid cells and non-malignant hematopoietic stem and progenitor cells. Technologies used to build newer CAR T may be able to simultaneously modify the allografts to limit off-target effects.

The pace of innovation in the adoptive immunotherapy space is accelerating, sparked by the success of both platforms; allo-HCT and CAR T. The ongoing research in both fields is routinely translated to one another and to other forms of investigational cellular therapies, providing strategies to manage complications, such as CRS, and increase accessibility with the prospect of UCART therapy. As the technologies evolve and new therapies emerge, the challenge will continue to be in synthesizing the data in reference to the specific disease and performance status of each patient in order to provide better and more tailored treatment for each individual.

## Author Contributions

SG wrote the manuscript. AG and JD reviewed and edited the manuscript in detail. All authors contributed to the article and approved the submitted version.

## Funding

NCI/NIH P50CA171963 Leukemia SPORE (JD), NIH/NCI: R35CA210084 NCI Outstanding Investigator Award (JD); Childrens Discovery Institute (JD).

## Conflict of Interest

JD: Honorarium Incyte; Board of Directors, Rivervest; research support Macrogenics, Equity/Ownership Magenta Therapeutics and WUGEN. AG: Consulting or advisory role and honoraria with Kite, a Gilead Company, and consulting and advisory role with Amgen, Atara, Wugen, and Celgene/BMS. SG: Consulting, Wugen.

The handling editor declared a past co-authorship with one of the authors JD.
